# A new simulated annealing algorithm for simultaneous open-pit and waste dump scheduling in mining complexes

**DOI:** 10.1371/journal.pone.0333606

**Published:** 2025-10-07

**Authors:** Jingsi Lin, Mohammad Waqar Ali Asad, Erkan Topal, Ping Chang

**Affiliations:** Western Australian School of Mines: Minerals, Energy, and Chemical Engineering, Curtin University, Carlisle, Australia; Aksum University, ETHIOPIA

## Abstract

Production scheduling models for open-pit mining complexes determine the optimal sequence for extracting mining blocks while adhering to technical and operational constraints. Although various mathematical models are available in the literature, solving them for large-scale operations remains computationally intensive. This challenge becomes more complex when models aim to simultaneously schedule block extraction and waste dumping, as this introduces additional variables and constraints, further complicating the problem. This paper presents a novel Simulated Annealing (SA)-based algorithm as a solution method for the simultaneous optimisation of block extraction and waste dumping sequences in open-pit mining complexes. The proposed approach incorporates an innovative variable reduction technique and a heuristic for generating reliable initial solutions. Numerical results show that the proposed algorithm achieves an NPV within 6.08% of the exact solution derived through a commercial solver while reducing the runtime by 82%. More importantly, for relatively larger instances that commercial solver fails to resolve, the proposed method maintains robust performance and delivers high-quality solutions within reasonable computational times.

## 1. Introduction

A mixed-integer programming (MIP)–based production scheduling model for an open-pit mining complex determines the optimal extraction sequence for mining blocks and the corresponding flow of valuable (ore) and waste materials across the supply chain, which includes multiple mines, processing facilities, stockpiles, and waste dumps, over a defined planning horizon. The MIP model incorporates geological, economic, and operational inputs. The geological input is a three-dimensional orebody block model comprising thousands of equal-sized blocks, each characterized by attributes such as mineral grade, rock type, and material density. The primary objective of the MIP model is to maximise the net present value (NPV) of future cash flows while satisfying operational constraints related to mining, processing, stockpiling, and waste dumping capacities. Additionally, it adheres to mining block precedence constraints, ensuring that all overlying blocks are removed before accessing blocks at lower levels within the three-dimensional orebody model. Therefore, the model structure closely aligns with a broader class of problems known as the precedence-constrained knapsack problem [1]. Given the large number of mining blocks and time periods, this becomes a strong NP-hard problem, posing significant computational challenges [2,3].

Given its complexity and significant impact on the economic success of mining projects, production scheduling models for open-pit mining have been extensively studied over the years. A crucial preliminary step involves applying pseudo-flow algorithms, which exclude time factors and resource constraints to define a maximum closure (ultimate pit limit) and identify the subregion or subset of the orebody for subsequent production scheduling [4–6]. As a result, production scheduling typically begins with the block model constrained within the ultimate pit limit, reducing both problem size and computational demands. Caccetta and Hill [7] confirms the optimality of this workflow within the mining industry. Despite this data reduction, the problem remains computationally complex, prompting the development of various methodologies for addressing large-scale instances over time. A review of the literature reveals that solution approaches to managing complexity generally falls into four main categories: model decomposition, block aggregation, metaheuristics, and component decoupling.

Model decomposition involves dividing a large, complex optimization problem into smaller, more manageable subproblems, each addressing a specific aspect of the original model, which can then be solved independently or in coordination. The first attempt was by Johnson [8], who applied Dantzig-Wolfe decomposition to break the linear programming model into a master problem and multiple subproblems, enabling efficient solution. However, only a few dozen blocks were used to demonstrate the concept. Chicoisne, Espinoza [9] introduced the critical multiplier algorithm, a novel decomposition method, alongside a TopoSort heuristic, to solve the LP relaxation of the production scheduling problem (C-PIT), while handling only a single resource constraint. Caccetta and Hill [7] developed a customised branch-and-cut algorithm combined with LP relaxation to obtain a tighter upper bound, which was further improved through heuristic methods. Nevertheless, they observed that convergence deteriorated as the problem size increased. Bienstock and Zuckerberg [10] extended the study of LP relaxation by accommodating arbitrary numbers of resource constraints (side constraints), significantly improving computational efficiency. Lambert and Newman [11] combined a sliding time window heuristic with the Lagrangian relaxation of resource constraints to solve instances with up to 25,000 blocks to near- optimality. More recently, Chatterjee and Dimitrakopoulos [12] proposed a variable reduction technique using Lagrangian relaxation to sequentially solve subproblems for each period. Nonetheless, Lagrangian relaxation suffers from drawbacks such as the duality gap and the inability to guarantee feasible solutions to the original problem [13,14].

Alternative approaches to tackling open-pit production scheduling problems have focused on aggregation methods, where blocks, periods, or destinations are grouped to reduce model size. Although aggregation can introduce suboptimality by losing operational detail, it enables large-scale problems to be solved using exact or heuristic-based methods. Ramazan [15] proposed a so-called fundamental tree algorithm (FTA), which identifies the smallest units of blocks with positive values that can be aggregated into “trees”, thereby reducing the number of integer variables. Boland, Dumitrescu [16] introduced a block aggregation procedure ensuring that aggregated block bins satisfy precedence requirements, which enables the solution of problems with nearly 10,000 blocks. Tabesh and Askari-Nasab [17] developed an agglomerative hierarchical clustering algorithm based on attributes such as rock type, grade, and block distance. Jélvez, Morales [18] presented a reblocking method by specifying aggregation sizes and recalculating block coordinates. Mai, Topal [19] expanded on Ramazan [15] by developing a new aggregation algorithm, known as the TopCone algorithm, which sets a minimum number of blocks per group to enhance variable reduction. While block aggregation substantially reduces problem size, the inaccurate representation of block attributes often leads to poor scheduling accuracy, rendering the solution far from practical [20]. Beyond block aggregations, Nancel-Penard, Morales [21] recently designed a recursive time aggregation-disaggregation (RAD) heuristic, assuming hat each block can be extracted within two consecutive periods. They defined meta-periods and recursively decomposed the integer program into two non-overlapping meta-periods until only a single period remained.

Despite advancements in decomposition and aggregation techniques, solving real-sized problems continues to incur high computational costs, particularly when using exact methods. The main limitation of exact approaches is their poor scalability, as the exponential growth in problem complexity renders them computationally intractable for large-scale instances [22,23]. To address this, a range of more computationally efficient metaheuristic algorithms has been developed. Metaheuristics are high-level algorithmic frameworks designed to enhance heuristic performance in solving complex optimisation problems [24]. Common metaheuristics applied in mine planning include simulated annealing (SA), particle swarm optimisation (PSO), genetic algorithm (GA), ant colony optimisation (ACO), firefly algorithm (FA), bat algorithm (BA), differential evolution (DE), tabu search (TS), and variable neighbourhood search (VNS) [25].

Moosavi, Gholamnejad [26] addressed the block sequencing problem using Lagrangian relaxation, incorporating mining and processing capacity constraints and grade blending into the objective function. Departing from conventional sub-gradient techniques, they applied a GA algorithm to update the Lagrangian multipliers. Khan and Niemann-Delius [27] adapted the continuous PSO algorithm for block extraction sequencing, introducing normalisation, penalty mechanisms, and a multi-start strategy to ensure solution feasibility and accelerate convergence. Similarly, Shishvan and Sattarvand [28] restructured the problem as an optimal depth determination problem and employed ACO for solution construction. Danish, Khan [29] proposed a variant of SA for production scheduling, incorporating a stockpile heuristic that swaps a block with one from the stockpile if its grade is lower than the average grade of material in the stockpile. Alipour, Khodaiari [22] applied GA to block scheduling problems and achieved less than a 5% optimality gap compared to exact methods, although their approach was limited to smaller problem sizes.

Several studies have explored the use of metaheuristics for the stochastic version of the problem, incorporating geological and economic uncertainties. For instance, Lamghari and Dimitrakopoulos [30] proposed a long-term memory strategy within a pure TS metaheuristic, demonstrating its robustness on a large-scale open-pit mine production scheduling problem involving up to 40,000 blocks, while accounting for metal uncertainty. This approach was further extended by Senécal and Dimitrakopoulos [31], who introduced multiple processing streams, including stockpiles and various processing destinations, and implemented a parallelised multi-neighbourhood TS to improve neighbourhood search efficiency. Paithankar and Chatterjee [32] applied maximum flow algorithm for block extraction sequencing under the financial uncertainty, with arc weights optimised using a GA. Comparative studies have also emerged, such as those by Goodfellow and Dimitrakopoulos [33], Goodfellow and Dimitrakopoulos [34], who integrated SA, PSO, and DE within a global optimisation framework for a mining complex modelled as a two-stage stochastic integer program. The hybrid algorithms delivered higher NPVs than standalone SA, however, at the cost of significantly longer computation times. Similarly, Tolouei, Moosavi [35] compared PSO, FA, and BA for block extraction sequencing under grade uncertainty. Various objective functions have also been explored. For example, Goodfellow and Dimitrakopoulos [36] applied SA to minimise the average deviation from a target tonnage across geological simulations when optimising pushback designs. In addition to using single or multiple metaheuristics, Lamghari and Dimitrakopoulos [37] proposed a hyper-heuristic framework to select or generate the most suitable heuristics or meta-heuristics for these problems, with or without stockpiling options. Further studies in this domain can be found in Lamghari and Dimitrakopoulos [30], Mokhtarian Asl and Sattarvand [38], Saliba and Dimitrakopoulos [39], Tolouei, Moosavi [40], and Yaakoubi and Dimitrakopoulos [41], with Silva, de Souza [42] providing a comprehensive overview of these solution methods.

Previous studies discussed thus far focus exclusively on the sequencing of mining block extraction and material flow within the supply chain of a mining complex. However, integrating block extraction with waste dump scheduling is essential, as waste haulage can account for up to 50% of operating costs [43]. An ore-oriented strategy without careful planning of waste dumping may result in significant additional haulage expenses. Beyond the financial implications, neglecting a detailed dumping schedule poses ecological risks, especially when mismanaging potentially acid-forming (PAF) waste. The generation of acid mine drainage (AMD) from PAF waste poses a serious environmental threat, contaminating water and soil and impacting health [44,45]. Moreover, AMD treatment also incurs high costs [46,47]. Therefore, a detailed waste scheduling plan is crucial for effective PAF waste management.

In this context, Williams, Topal [48] developed a mixed-integer linear programming (MILP) model to minimise waste haulage costs and ensure PAF is encapsulated by non-acid-forming (NAF) waste. Li, Topal [43], Li, Topal [49] further enhanced this by introducing a three-dimensional dump design with precedence constraints for multi-lift dumping, but their method separates extraction sequencing from waste allocation, using a two-step procedure: first, block extraction sequence is determined, then waste is allocated optimally within dump cells for both PAF and NAF waste. As such, they do not represent a fully integrated framework.

Fu, Asad [50] addressed this gap by proposing a simultaneous optimisation of block extraction and waste dump scheduling using the same dump design as Li, Topal [43]. Lin, Asad [51] extended this approach to a more complex scenario involving multiple mines, processes, stockpiles, and dumps within a mining complex. While both studies achieved a close optimality gap (less than 5%), their reliance on exact solution methods and the large number of variables limit their applicability to small-scale problems. Beyond PAF encapsulation, Vaziri, Sayadi [52] proposed a waste blending approach using a mixed-integer programming (MIP) model for short-term production scheduling. This model blended NAF, acid-neutral, and PAF waste to minimise AMD generation. For a comprehensive review of recent advances in waste management, refer to Das, Topal [53].

Based on the literature review, it is evident that decomposition and aggregation strategies, along with the absence of integrated waste dumping optimisation, can undermine both the solution feasibility and optimality. Furthermore, the NP-hard nature of large-scale problems restricts the practical application of commercial solvers. In response to these challenges, this paper develops a new simulated annealing (SA)-based framework designed to manage computational complexity while incorporating operational realism. As highlighted by Nikolaev and Jacobson [54], there are two primary directions for accelerating convergence toward high-quality solutions in SA: (i) problem-specific strategies, such as designing tailored objective functions and developing effective neighbourhood structures; and (ii) generic strategies, including modifications to the acceptance probability function and optimisation of the cooling schedule.

In line with advancing problem-specific strategies, the contributions of this paper are threefold:

1. Integration of progressive waste-dumping with PAF encapsulation: to our knowledge, this is the first SA framework that explicitly incorporates progressive waste-dump scheduling under PAF encapsulation requirements. While this integration increases computational complexity, it is effectively managed through a novel variable reduction strategy and a destination-based perturbation scheme (Sections 2.2 and 3.2.2) leading to a reduction in waste rehandling and an enhancement in NPV.
2. Development of diverse perturbation mechanisms: conventional SA implementations for mine scheduling apply perturbations that shift candidate blocks earlier or later in the schedule (See Saliba and Dimitrakopoulos [39], Leite and Dimitrakopoulos [55], Albor Consuegra and Dimitrakopoulos [56], Dimitrakopoulos [57], Montiel and Dimitrakopoulos [58], Kumral [59], Danish, Khan [60]). While effective, such approaches tend to converge to local optima [61]. By contrast, this study introduces three complementary perturbations (mining-based, destination-based, and grade-based), designed to satisfy block precedence, mining, and processing capacity constraints. Although diverse perturbations have been explored previously for stochastic models (e.g., Montiel and Dimitrakopoulos [61] and Montiel, Dimitrakopoulos [62]), these remain restricted to block-level operations. In contrast, we propose partial-block swapping for a partial mining model for mining complexes (Lin, Asad [51]), thereby expanding the search space and improving solution diversity.
3. A heuristic for reliable initialisation: this study introduces an innovative heuristic for generating a reliable initial feasible solution that accelerates convergence to high-quality solutions in the proposed SA algorithm.

The application of these procedures to an operational gold mining complex demonstrates the effectiveness of the proposed method in jointly optimising block extraction and detailed waste dumping schedules, particularly in addressing the environmental risks associated with Acid Mine Drainage (AMD). This is, to the best of our knowledge, the first study to implement an SA-based approach that integrates both variable reduction and an initial solution heuristic for the simultaneous optimisation of mining complexes with detailed waste dump scheduling.

The remainder of the paper is organised as follows: Section 2 introduces the mathematical formulation, along with the variable reduction procedure and the initialisation heuristic. Section 3 outlines the solution improvement phase using the SA algorithm. Section 4 presents and discusses the results of case studies, followed by Section 5, which concludes the paper.

## 2. Mathematical formulation

The mathematical formulation in this section relies on the open-pit mining complex framework presented in Lin, Asad [51]. This framework considers dynamic cut-off grades for material classification into ore and waste, i.e., as opposed to pre-classification of ore and waste based on breakeven cut-off grade implemented in previous studies, the formulation is structured to dynamically define the cut-off grade and accordingly allocate the extracted materials from the multiple mines to processing streams, stockpiles, and waste dumps. The stockpiles in this framework constitute multiple grade bins, which facilitates tracking of the quality and quantity of material sent to and retrieved from the stockpiles as practiced in the industry. Similarly, the waste material is allocated to equal-sized dump cells within the waste dumps with an allowance or flexibility for multi-lift dumping coupled with a dump cell precedence requirement, i.e., a dump cell located in an upper lift may be filled only if the dump cells located in the lower lift are filled. In addition, for full encapsulation of the PAF waste, some dump cells may accept NAF waste only, with the possibility to supply NAF waste from the waste stockpiles if NAF waste may not be supplied from the pits. With this framework for the mathematical formulation, all sets and indices, parameters, and decision variables are defined as outlined below.

Sets and indices:

**Table:** 

t∈T	set of periods or years
$b\in B_{m}$	set of blocks in mine m∈M
$d\in D_{w}$	set of dump cells in waste dump w∈W
$\Pi_{b}$ and $\Pi_{d}$	set of direct precedent blocks for block b or direct precedent dump cells for dump cell d
i∈M∪P∪S∪W	set of locations including mines M, processing plant P, stockpiles S, and waste dump W. Among these, ${\text{A}}_{\text{i}}=\left\{M\cup S\right\}$ denotes set of source locations i that send materials, while ${\text{B}}_{i}=\left\{P\cup S\cup W\right\}$ denotes set of target locations i that receive materials
Parameters:	
r	interest rate (%)
$U_{it}$	Upper capacity limit of location i in period t, i∈M∪P
$C_{i}$	Upper capacity limit of location i, i∈S∪W
${\underset{―}{G}}_{pt}$	minimum ore grade required at process p in period t (% content of metal or grams of metal per tonne or troy ounces of metal per tonne, etc.)
$q_{bm}$	total reserves in block b of mine m (tonne of material)
$\sigma_{i}$	material density for materials in source location i
ρ	material swell factor (%)
$v_{ij}$	Undiscounted economic value of material supplied from source location i to target location j. These values are location-based, i.e., $v_{ij}=\left(p^{\prime }-r^{\prime }\right)g_{i}r_{ij}-c_{i}-h_{ij}d_{ij}-c_{ij}$, if the target location jϵP, otherwise, $v_{ij}=-c_{i}-h_{ij}d_{ij}$, where $p^{\prime }$ is the metal price; $r^{\prime }$ is the metal refining cost; $g_{i}$ is material grade from source locations i; $r_{ij}$ is the recovery rate of materials from location i to location j; $c_{i}$ is the mining cost if location iϵM, or rehandling cost if location iϵS; $h_{ij}$ is the haulage cost from location i to location j; $d_{ij}$ is the distance from location i to location j; and $c_{ij}$ is the processing cost from location i to location j.
Decision Variables:	
$x_{ijt}\;=$	quantity of material supplied from source location i to target location j in period t
$y_{bmt}=$	$\left\{ \begin{array}{cc} \text{1} & \text{if all blocks overlying block}\;b\;\text{in mine}\;m\;\text{are mined out in periods to}\;t\\ \text{0} & \text{otherwise} \end{array}\right.$
$y_{dwt}=$	$\left\{ \begin{array}{cc} \text{1} & \text{if all precedent cells below cell }d\;\text{in dump }w\;\text{are filled in periods to }t\\ \text{0} & \text{otherwise} \end{array}\right.$

### 2.1 A mixed integer linear programming (MILP) model

Objective function:


$$\begin{array}{c} Max\sum {\mathrm{\backslash nolimits}}_{i\in {\text{A}}_{i}}\sum {\mathrm{\backslash nolimits}}_{j\in {\text{B}}_{j}}\sum {\mathrm{\backslash nolimits}}_{t\in T}\frac{v_{ij}}{(\text{1}+r{)}^{t}}x_{ijt} \end{array}$$ (1)

Subject to:


$$\begin{array}{c} \sum {\mathrm{\backslash nolimits}}_{j\in {\text{B}}_{j}}\sum {\mathrm{\backslash nolimits}}_{t\in T}x_{bjt}\leq q_{bm},\forall b\in B_{m},m\in M \end{array}$$ (2)

$$\begin{array}{c} \sum {\mathrm{\backslash nolimits}}_{b\in B_{m}}\sum {\mathrm{\backslash nolimits}}_{j\in {\text{B}}_{j}}x_{bjt}\leq U_{mt},\forall m\in M,t\in T \end{array}$$ (3)

$$\begin{array}{c} \sum {\mathrm{\backslash nolimits}}_{i\in {\text{A}}_{\text{i}}}x_{ipt}\leq U_{pt},\forall p\in P,t\in T \end{array}$$ (4)

$$\begin{array}{c} \sum {\mathrm{\backslash nolimits}}_{b\in B_{m}}\sum {\mathrm{\backslash nolimits}}_{m\in M}\sum {\mathrm{\backslash nolimits}}_{t^{\prime }=\text{1}}^{t}x_{bit^{\prime }}-\sum {\mathrm{\backslash nolimits}}_{j\in {\text{B}}_{j}}\sum {\mathrm{\backslash nolimits}}_{t^{\prime }=\text{1}}^{t}x_{ijt^{\prime }}\geq \text{0},\forall i\in S,t\in T \end{array}$$ (5)

$$\begin{array}{c} \rho \sum {\mathrm{\backslash nolimits}}_{i\in {\text{A}}_{i}}\sum {\mathrm{\backslash nolimits}}_{t\in T}\frac{x_{idt}}{\sigma_{i}}\leq C_{d},\forall d\in D_{w},w\in W,t\in T \end{array}$$ (6)

$$\begin{array}{c} \rho \frac{\sum_{i\in {\text{A}}_{i}}\sum_{d\in D_{w}}\sum_{t\in T}x_{idt}}{\sigma_{i}}\;=\sum {\mathrm{\backslash nolimits}}_{d\in D_{w}}C_{d},\forall w\in W \end{array}$$ (7)

$$\begin{array}{c} \sum {\mathrm{\backslash nolimits}}_{u\in \Pi_{b}\;}\sum {\mathrm{\backslash nolimits}}_{j\in {\text{B}}_{j}}\sum {\mathrm{\backslash nolimits}}_{t^{\prime }=\text{1}}^{t}x_{ujt^{\prime }}\;\geq y_{bmt}\sum {\mathrm{\backslash nolimits}}_{u\in \Pi_{b}\;}q_{u},\forall b\in B_{m},\;m\in M,t\in T \end{array}$$ (8)

$$\begin{array}{c} \sum {\mathrm{\backslash nolimits}}_{j\in {\text{B}}_{j}}\sum {\mathrm{\backslash nolimits}}_{t^{\prime }=\text{1}}^{t}x_{bjt^{\prime }}\;\leq y_{bmt}q_{bm},\forall b\in B_{m},\;m\in M,t\in T \end{array}$$ (9)

$$\begin{array}{c} \rho \sum {\mathrm{\backslash nolimits}}_{o\in \Pi_{d}}\sum {\mathrm{\backslash nolimits}}_{i\in {\text{A}}_{i}}\sum {\mathrm{\backslash nolimits}}_{t^{\prime }=\text{1}}^{t}\frac{x_{iot^{\prime }}}{\sigma_{i}}\;\geq y_{dwt}\sum {\mathrm{\backslash nolimits}}_{o\in \Pi_{d}}C_{o},\forall d\in D_{w},w\in W,t\in T \end{array}$$ (10)

$$\begin{array}{c} \rho \sum {\mathrm{\backslash nolimits}}_{i\in {\text{A}}_{i}}\sum {\mathrm{\backslash nolimits}}_{t^{\prime }=\text{1}}^{t}\frac{x_{idt^{\prime }}}{\sigma_{i}}\leq y_{dwt}C_{d},\forall d\in D_{w},w\in W,t\in T \end{array}$$ (11)

$$\frac{\sum_{i\in {\text{A}}_{i}}x_{ijt}g_{i}}{\sum_{i\in {\text{A}}_{i}}x_{ijt}}{\geq \underset{―}{G}}_{pt},\;\forall j\in P,t\in T$$ (12)

$$x_{ijt}\geq \text{0},y_{bmt},y_{dwt}\in \left\{\text{0},\;\text{1}\right\},\forall i\in M\cup S,j\in P\cup S\cup W,i\neq j$$ (13)

The objective function (1) is the sum of the discounted values of materials from pits to processing streams, stockpiles and waste dumps, and from stockpiles to processing streams and waste dumps.

Equation (2) represents the reserve constraint that forces the quantity of material in a block b sent to processing streams, stockpiles and waste dumps over the planning horizon is no more than the quantity of available material in the block. Equations (3) and (4) ensure mining and processing rates remain with mining and processing capacities per period. Equation (5) enforces an inventory balance requirement for stockpile bins. Note that no material will be retrieved from the stockpiles in period 1 (i.e., $\sum_{j\in \text{B}(j)}\sum_{t^{\prime }=\text{1}}^{t}x_{ijt^{\prime }}=\text{0}$ if t=1). Equations (6) and (7) enforce the dumping capacity constraint and PAF encapsulation requirements, respectively. Equations (8) and (9) are the block precedence constraints that ensure the complete mining of all overlying blocks before a block below is considered for extraction. Similarly, Equations (10) and (11) represent waste dumping precedence rules for a multi-lift dumping strategy. Equation (12) stands for the grade blending requirements guaranteeing that the material supplied to the processing streams has a weighted average grade of at least ${\underset{―}{G}}_{pt}$ per period. Equation (13) defines the nature of decision variables.

### 2.2 Variable reduction technique

Due to the NP-hard nature of the problem, the computational time grows exponentially, making it impractical to solve without employing relaxation or decomposition strategies. For instance, the formulation presented in Section 2.1 for a mining complex results in $\left(\sum_{m}B_{m}+\sum_{w}D_{w}\right)\times T$ binary variables, and $\sum_{m}B_{m}\times \left(P+S+\sum_{w}D_{w}\right)\times T$ continuous variables, and $\sum_{m}B_{m}+\left(M+\text{2}P+S+\text{2}\sum_{m}B_{m}+\text{2}\sum_{w}D_{w}\right)\times T$ linear constraints. In a typical large-scale case with $\sum_{m}B_{m}=\text{1}\text{0}\text{0},\text{0}\text{0}\text{0}$ blocks and T=10 periods, this complexity often leads to memory overflow or excessive computational demands even for commercial solvers like IBM ILOG CPLEX as well as the methods proposed in previous studies [7,63]. To address this challenge, we detail the proposed technique for variable reduction before applying the SA algorithm in this section. The strategy aims to identify blocks and dump cells that cannot be extracted or filled due to resource constraint violation in each period so that the associated binary and continuous variables are eliminated. To elaborate, the following two preliminaries are first introduced.

**Preliminary 1.** The mining capacity serves as the primary bounding factor in determining the block extraction sequence, because the overproduction of low-grade or medium-grade ore in a given period may be stored in stockpiles for future processing, and accordingly, the processing capacity is not a bottleneck resource type in this problem.

**Preliminary 2.** The quantity of waste material from the extractable blocks defined through Preliminary 1 becomes a bottleneck for waste dumping schedules given the restricted number of available dump cells in each period.

The following steps outline the procedure for reducing the number of variables.

Step 1: Calculate the ultimate pit limit (UPL) using the maximum-flow algorithm [64].

Step 2: Within the UPL, inverted cones are constructed as $IC=\left\{{IC}_{\text{1}},{IC}_{\text{2}},\cdots ,{IC}_{N}\right\}$, which are spatially continuous and arranged in ascending order based on the number of blocks included. Each cone consists of a base block $b_{k}$ and all its preceding blocks $\left\{pred\left(b_{k}\right)\right\}$, such that ${IC}_{i}=b_{i}\cup \left\{pred\left(b_{i}\right)\right\}$. In parallel, a corresponding set of upright cones, $RC=\left\{{RC}_{\text{1}},{RC}_{\text{2}},\cdots ,{RC}_{N}\right\}$, associated with each base block $b_{k}$ and all its succeeding blocks, is computed in parallel, such that ${RC}_{k}=b_{k}\cup \left\{sucd\left(b_{k}\right)\right\}$.

**Fig 1:**
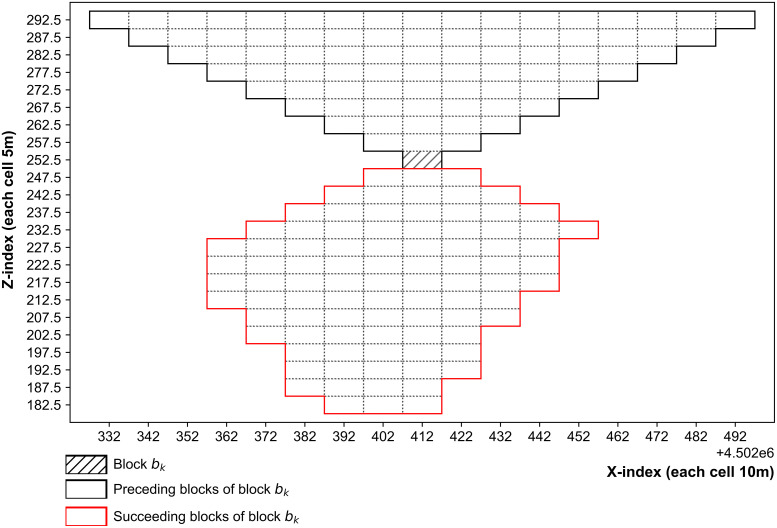
An illustration of preceding and succeeding sets of blocks for a base block $b_{k}$.

More specifically, block precedence relationships follow the 1:5 pattern [64] implemented in most commercial mining software [65], i.e., for a base block $b_{k}$ located at a mining level or bench below surface, a set of five blocks at mining level or bench immediately above must be extracted to maintain a 45 degrees of slope angle for a geotechnically stable operation. Logically, this predecessor requirement multiplies if base block $b_{k}$ is located several levels or benches below the surface. Conversely, by applying the identical pattern, upright cones represent the sets of blocks that must succeed the base block. Fig 1 illustrates a 2D example of a base block $b_{k}$ along with its associated predecessors and successors in a real case.

By iteratively checking whether the total quantity of each inverted cone exceeds the mining capacity limit, the earliest mining period for each block can be identified. Algorithm 1 describes the procedure for detecting the earliest mining period for each block. Consequently, the earliest mining period for each block $b_{i}$ is determined as $t_{b_{k}}^{earliest}=\underset{t}{argmin}\left(y_{b_{k}mt}=\text{1}\right)$.

**Algorithm 1. *Pseudocode of earliest mining period detection***

1. set variables $y_{bmt}=\text{0}$, ∀b∈UPL; set all upright cones ${RC}_{i}=\emptyset$ where ${RC}_{i}\in \;RC$

2. **While** t≤|T| **do**

3.  **While** k≤N **do**

4.   **If** $b_{k}\in {⋃}_{k^{\prime }\leq k}{RC}_{k^{\prime }}$ **then**

5.    continue

6.   **If** $\sum_{j\in pred\left(b_{k}\right)}q_{j}<U_{mt}$ **then**

7.    update $y_{bmt}\leftarrow \text{1}$, $\forall b\in {IC}_{k}$

8.   **Else** update ${RC}_{k}\leftarrow \text{1}\left({RC}_{i}\;=\;\emptyset \right){RC}_{k}+\text{1}\left({RC}_{k}\;\neq \;\emptyset \mathrm{\backslash rightleft}(\left\{b_{k}\right\}\cup \left\{sucd\left(b_{k}\right)\right\}\right)$

9.   **If** ${⋃}_{k^{\prime }\leq k}\left({IC}_{k^{\prime }},{RC}_{k^{\prime }}\right)=UPL$ **then**

10.    update $y_{bmt}\leftarrow max\left\{y_{bmt^{\prime }}|t^{\prime }\in [\text{0},t]\right\}$, $\forall b\in {⋃}_{k}\left({IC}_{k},{RC}_{k}\right)$

11.    update ${IC}_{k}\leftarrow {IC}_{k}|\left\{y_{bmt}\neq \text{1}\right\}\;$

12.    break

13. **End**

Step 3: Given the extractable blocks $b\in B_{m}^{\prime }$ identified in each period, the problem of establishing the earliest period for filling each dump cell while considering dumping capacity, dumping precedence, and waste material flow balance constraints is now addressed. This step helps decrease the binary variables representing the dump cell filling status $\left(y_{dwt}\right)$ and the corresponding continuous variables $\left(x_{bdt}\right)$ that define material flow from blocks to dump cells in each period. Naturally, dump cells get filled sooner if higher quantity of waste is sent to the waste dump. Therefore, as a strategy, maintaining a higher cut-off grade classifies more blocks within $B_{m}^{\prime }$ as waste and then moves this material to the waste dumps and waste stockpiles ensuring that the calculated earliest period for filling the dump cell in this step is sooner than the optimal plan. In addition, to reduce the risk of AMD, the period index t is used as a multiplier to the binary variables representing the dump cell filling status $\left(y_{dwt}\right)$ (Equation (14)), thereby encouraging the deferral of PAF material dumping as long as operational constraints permit. The formulation that ensures implementation of step 3 is provided as follows.

Objective function:


$$\begin{array}{c} Min\sum {\mathrm{\backslash nolimits}}_{d\in D_{w}}\sum {\mathrm{\backslash nolimits}}_{w\in W}\sum {\mathrm{\backslash nolimits}}_{t\in T}ty_{dwt} \end{array}$$ (14)

Subject to Constraints (5)-(7), (10)-(11), (13) and a new constraint below.


$$\sum_{j\in {\text{B}}_{j}}\sum_{t\in T}x_{bjt}=q_{bm},\forall b\in B_{m}^{\prime },m\in M\;$$ (15)

The objective function (14) minimises the sum of the earliest possible periods where each dump cell is available for dumping. Equation (15) guarantees that the quantity of material supplied from a block to the waste dump or waste stockpiles across all periods is equal to the total quantity of available material in that block.

The above formulation involves relatively fewer blocks and dump cells per period, allowing it to be efficiently solved using exact methods within a reasonable timeframe. The exact method applied in this study is the branch-and-cut algorithm implemented using the CPLEX solver. After solving the problem, the earliest filling time of each dump cell is determined as the minimum time period in which all its precedent dump cells have been filled, expressed as $\underset{t}{argmin}\left(y_{dwt}=\text{1}\right)$.

### 2.3 Initial feasible solution

With the reduced size of the problem, the initial solution is derived using the “decompose-and-combine” concept [66] with an innovative heuristic approach. To elaborate, the problem is broken down into a series of smaller sub-problems, each associated with a specific period t. The sub-problems are then solved sequentially, starting from period t=1 and continuing until t=|T|. While the decomposition process resembles the stochastic version presented in Lamghari and Dimitrakopoulos [67], this work expands by integrating a detailed waste dumping schedule component into the formulated sub-problem.

The sub-problem (SP) for period t, denoted as ${SP}_{t}$, determines:

- The set of blocks $b\in \Lambda_{b}^{t}$ to be extracted and dump cells $d\in \Lambda_{d}^{t}$ to be filled in period t.
- The quantity of material from extracted blocks and the materials retrieved from stockpiles for delivery to their respective processing destinations $\left(x_{ij}^{*},i\in {\text{A}}_{i},j\in \text{B}\right)$. In this regard, the following five parameters are introduced to track the block mining and material transportation status from previous periods.

**Table:** 

${BQ}_{b}^{t}$	the cumulative quantities of material in block b at the beginning of period t, where ${BQ}_{b}^{t}={BQ}_{b}^{t-\text{1}}+\sum_{i\in A_{i}}\sum_{j\in B_{j}}x_{ij}^{*},\forall t>\text{1}$
${AQ}_{s}^{t}$	the quantity of materials stored in stockpile s at the beginning of period t, where ${AQ}_{s}^{t}=\left\{ \begin{array}{cc} {AQ}_{s}^{t-\text{1}}+\sum_{b\in \Lambda_{b}^{t}}x_{bs}^{*}-\sum_{p\in P}x_{sp}^{*} & t>\text{1}\;and\;s\in ore\;stockpiles\\ {AQ}_{s}^{t-\text{1}}+\sum_{b\in \Lambda_{b}^{t}}x_{bs}^{*}-\sum_{d\in \Lambda_{d}^{t}}x_{sd}^{*} & t>\text{1}\;and\;s\in waste\;stockpiles \end{array}\right.\;$
${DV}_{d}^{t}$	the cumulative volume of waste materials in dump cell d at the beginning of the period t, where ${DV}_{d}^{t}={DV}_{d}^{t-\text{1}}+\rho \frac{\sum_{i\in {\text{A}}_{i}}x_{id}^{*}}{\sigma_{i}},\forall t>\text{1}$
${PQ}_{bj}^{t}$	the cumulative quantity of materials in precedence blocks of block b, all included in $\Lambda_{b}^{t}$, that have been mined and transported to destination j at the beginning of period t, where ${PQ}_{bj}^{t}={PQ}_{bj}^{t-\text{1}}+\sum_{b\in \Pi_{b}⋂\Lambda_{b}^{t}}x_{bj}^{*},\forall t>\text{1}$
${PV}_{id}^{t}$	the cumulative volume of precedent dump cells of dump cell d, all included in $\Lambda_{d}^{t}$, that have been filled with waste materials from source location i at the beginning of period t, where ${PV}_{id}^{t}={PV}_{id}^{t-\text{1}}+\sum_{d\in \Pi_{d}⋂\Lambda_{d}^{t}}x_{id}^{*},\forall t>\text{1}$

Note that all the five parameters above are equal to 0 when solving the first sub-problem (i.e., period t=1). Therefore, the ${SP}_{t}$ can be formulated as follows.


$$\begin{array}{c} Max\sum {\mathrm{\backslash nolimits}}_{i\in {\text{A}}_{i}}\sum {\mathrm{\backslash nolimits}}_{j\in {\text{B}}_{j}}v_{ij}x_{ij} \end{array}$$ (16)

Subject to:


$$\begin{array}{c} \sum {\mathrm{\backslash nolimits}}_{j\in \text{B}(j)}x_{bj}+{BQ}_{b}^{t}\leq q_{bm},\forall b\in \Lambda_{b}^{t},m\in M \end{array}$$ (17)

$$\begin{array}{c} \sum {\mathrm{\backslash nolimits}}_{b\in \Lambda_{b}^{t}}\sum {\mathrm{\backslash nolimits}}_{j\in \text{B}(j)}x_{bj}\leq U_{mt},\forall m\in M \end{array}$$ (18)

$$\begin{array}{c} \sum {\mathrm{\backslash nolimits}}_{i\in A_{i}}x_{ip}\leq U_{pt},\forall p\in P \end{array}$$ (19)

$$\begin{array}{c} {AQ}_{i}^{t}+\sum {\mathrm{\backslash nolimits}}_{b\in \Lambda_{b}^{t}}x_{bi}-\sum {\mathrm{\backslash nolimits}}_{j\in {\text{B}}_{j}}x_{ij}\geq \text{0},\forall i\in S \end{array}$$ (20)

$$\begin{array}{c} \sum {\mathrm{\backslash nolimits}}_{u\in \Pi_{b}⋂\Lambda_{b}^{t}}\sum {\mathrm{\backslash nolimits}}_{j\in {\text{B}}_{j}}x_{uj}+\sum {\mathrm{\backslash nolimits}}_{j\in {\text{B}}_{j}}{PQ}_{bj}^{t}\;\geq y_{bmt}\sum {\mathrm{\backslash nolimits}}_{u\in \Pi_{b}⋂\Lambda_{b}^{t}}q_{u},\forall b\in \Lambda_{b}^{t},\;m\in M \end{array}$$ (21)

$$\begin{array}{c} \sum {\mathrm{\backslash nolimits}}_{j\in {\text{B}}_{j}}x_{bj}\leq y_{bmt}\left(q_{bm}-\sum {\mathrm{\backslash nolimits}}_{b\in {⋃}_{t^{\prime }\leq t}\Lambda_{b}^{t^{\prime }}}{BQ}_{b}^{t}\right),\forall b\in \Lambda_{b}^{t},\;m\in M \end{array}$$ (22)

$$\begin{array}{c} \rho \frac{\sum {\mathrm{\backslash nolimits}}_{i\in A_{i}}\sum_{d\in \Lambda_{d}^{t}}x_{id}}{\sigma_{i}}+\sum {\mathrm{\backslash nolimits}}_{d\in {⋃}_{t^{\prime }\leq t}\Lambda_{d}^{t^{\prime }}}{DV}_{d}^{t}=\sum {\mathrm{\backslash nolimits}}_{d\in D_{w}}C_{dw},\forall w\in W,t=T \end{array}$$ (23)

$$\begin{array}{c} \rho \sum {\mathrm{\backslash nolimits}}_{i\in A_{i}}\sum {\mathrm{\backslash nolimits}}_{o\in \Pi_{d}⋂\Lambda_{d}^{t}}\frac{x_{io}}{\sigma_{i}}+\sum {\mathrm{\backslash nolimits}}_{i\in A_{i}}{PV}_{id}^{t}\;\geq y_{dwt}\sum {\mathrm{\backslash nolimits}}_{o\in \Pi_{d}⋂\Lambda_{d}^{t}}C_{o},\forall d\in \Lambda_{d}^{t},w\in W \end{array}$$ (24)

$$\begin{array}{c} \rho \sum {\mathrm{\backslash nolimits}}_{i\in A_{i}}\frac{x_{id}}{\sigma_{i}}\leq y_{dwt}\left(C_{d}-\sum {\mathrm{\backslash nolimits}}_{d\in {⋃}_{t^{\prime }\leq t}\Lambda_{d}^{t^{\prime }}}{DV}_{d}^{t}\right),\forall d\in \Lambda_{d}^{t},w\in W \end{array}$$ (25)

$$\frac{\sum_{i\in A_{i}}x_{ij}g_{i}}{\sum_{i\in A_{i}}x_{ij}}{\geq \underset{―}{G}}_{pt},\;\forall j\in P$$ (26)

$$x_{ij}\geq \text{0},y_{bmt},y_{dwt}\in \left\{\text{0},\;\text{1}\right\},\forall i\in M\cup S,j\in P\cup S\cup W,i\neq j$$ (27)

Equation (23) imposes the PAF encapsulation requirement and they are applicable only in the final mining period ensuring complete filling of the dump cells overlying PAF material. The rest of the formulation, including the objective function and remaining constraints, correspond to those presented in Section 2.1. To solve the sub-problem ${SP}_{t}$, we employ a higher-value-search heuristic (HVS), with the details provided below.

The HVS heuristic aims to find a set of blocks that meet the precedence relationships and resource constraints, while striving to achieve the higher economic value in each period. Note that the economic value of each block $v_{b}$ is determined by the highest value assigned to a processing destination, excluding stockpiles. Algorithm 2 describes steps of the HVS heuristic.

**Algorithm 2. *Pseudocode of HVS heuristic***

1. Construct the new set of inverted cones ${IC}^{t}=\left\{{IC}_{\text{1}}^{t},{IC}_{\text{2}}^{t},\cdots ,{IC}_{Nt}^{t}\right\}$ as introduced by Step 2 in Section 2.2; set $\Lambda_{b}^{t}=\emptyset$; set the economic value of each period $v^{t}=\text{0}$; set the remaining mining capacity ${RU}_{mt}=U_{mt}$; set the processing capacity ${RU}_{pt}=U_{pt}$

2. **While** t≤|T| **do**

3.  calculate the Ultimate Pit Limit (UPL) for each inverted cone ${IC}_{nt}^{t}$ and choose the inverted cone ${IC}_{max}^{t}$ with the largest UPL value $v_{upl}^{t}$. Sequentially insert the set of blocks $B_{k}^{t}=\left\{b_{\text{1}},\;b_{\text{2}},\cdots ,b_{k}\right\}$ in block mining sequence order into $\Lambda_{b}^{t}$, where $B_{k}^{t}\in {IC}_{max}^{t}$.

4. Update the economic value $v^{t}\leftarrow v^{t}+v_{upl}^{t}$; and update the remaining mining capacity ${RU}_{mt}\leftarrow {RU}_{mt}-\sum_{b\in \Lambda_{b}^{t}}q_{bm}$

5.  **If** $v_{upl}^{t}<\text{0}$ **then**

6.   continue

7.  **If** the remaining mining capacity ${RU}_{mt}<\text{0}$ **then**

8.   update ${IC}_{nt}^{t}\leftarrow \left\{{IC}_{nt}^{t}|b\notin \Lambda_{b}^{t}\right\}$; and update ${IC}_{max}^{t}$, $B_{k}^{t}$, $\Lambda_{b}^{t}$ as in step 3

**9.**  **Else** remove blocks from the opposite direction one by one from the latest $B_{k}^{t}$ until the mining capacity constraint (Equation (18)) is satisfied.

**10.**  update parameters ${BQ}_{b}^{t}$ and ${PQ}_{bj}^{t}$ accordingly; update $y_{bmt}\leftarrow \text{1}\left(b{\in \Lambda }_{b}^{t}\right)+y_{bmt}$

11.  solve the waste dump scheduling problem with the objective of minimising haulage costs, i.e., Min $\sum_{i\in {\text{A}}_{i}}\sum_{j\in d_{w}}\sum_{w\in W}v_{id}x_{id}$, subjects to Equations (5)-(7), (10)-(11), (13), and (15), when period t<T. Update parameters ${DV}_{d}^{t}$ and ${PV}_{id}^{t}$ accordingly.

12.  update the remaining processing capacity ${RU}_{pt}\leftarrow {RU}_{pt}-\sum_{\underset{b\in \Lambda_{b}^{t}}{argmax}v_{p}}q_{bm}$

13.  **If** ${RU}_{pt}<\text{0}$ **then**

14.   sort those blocks in ascending order according to their grade. Rearrange low-to-medium blocks $R_{s}^{t}$ to stockpiles one by one until the remaining blocks can be accepted by processing plants.

15.  **If** t>1 **then**

16.   replace blocks in the last $v_{upl}^{t}$ intended to insert into $\Lambda_{b}^{t}$ with materials retrieved from ore stockpiles if $\sum \sum_{p}v_{sp}x_{sp}>v_{upl}^{t}$ for some or all of the materials in ore stockpiles.

17.  Update parameter ${AQ}_{s}^{t}$ accordingly.

18.  **If** t=|T| **then**

19.   calculate the volume of materials sent from waste stockpiles to the waste dump $x_{sd}$ to satisfy Equation (23).

**20. End**

**3 Solution Improvement with SA**

Simulated annealing (SA) is an optimisation algorithm designed to solve large combinatorial problems [68]. SA offers several advantages when applied to solve production scheduling problem for a mining complex. The foremost benefit comes from efficient handling of the precedence constraints as they are primary reason for making mine production scheduling problem NP-hard. In this context, the candidate blocks for swapping can be easily identified by checking the accessibility of their preceding and succeeding sets of blocks, represented as inverted and upright cones (Fig 1). In contrast, population-based algorithms such as PSO and GA are less flexible in handling precedence constraints, which makes solution updates more computationally intensive [59].

In addition, SA can seamlessly incorporate dynamic cut-off grade strategies by allowing block processing destinations to be swapped, thereby enhancing scheduling flexibility. SA also requires only a single initial solution, whereas population-based metaheuristics must generate diverse and feasible populations. For large-scale, highly constrained mining complexes, generating such populations can be challenging, while realising that the quality of the initial solutions critically influences overall algorithmic performance.

In mining applications, SA is often enhanced through modifications of the acceptance probability function [69], optimisation of the cooling schedule [59] and hybridisations with other metaheuristics [33,34,60]. While direct comparisons of SA with other approaches in mining are limited, evidence from other domains is relevant and informative. For instance, Chai, Li [70] compared GA, PSO, and SA for real-time task scheduling on chip-multiprocessors and concluded that SA offers the best computational efficiency. Panda [71] compared PSO and SA for the Traveling Salesman Problem (TSP) problem. PSO achieved the best solution quality, while SA was preferable when execution time was critical. These considerations collectively motivate the choice of SA applicable to large-scale mining complex optimisation for this study. The SA procedures are introduced briefly below.

Starting from the initial feasible solution $e_{\text{0}}$, SA explores the solution space e∈E by iteratively perturbing the current solution e and then generate a new candidate solution $e^{\prime }$ within its neighbourhood N(e). Improved solutions are always accepted, while worse ones are accepted with a probability based on change in objective value $\Delta =obj\left(e^{\prime }\right)-obj(e)$ and a temperature parameter $T^{sa}$. This acceptance probability follows the Metropolis criteria [72], as provided in Equation (28) for a maximisation problem.


$$p_{acc}=\left\{ \begin{array}{cc} \text{1} & if\;\Delta \geq \text{0}\\ {exp}^{\frac{\Delta }{T^{sa}}} & if\;\Delta <\text{0} \end{array}\right.\;$$ (28)

The temperature gradually decreases over iterations, leading to a reduced acceptance probability of worsening solutions. As a result, each iteration increasingly resembles a local search. In this context, a robust cooling schedule is crucial for updating the temperature $T^{sa}$, as it directly governs the balance between exploration and exploitation [73]. By adjusting the probability of accepting worse solutions, it effectively guides the algorithm towards discovering a globally better solution $e^{*}$. On the other hand, a termination criterion is evaluated at each iteration to decide when to stop the SA process and return the best solution found so far. In summary, the key components that define SA include the initial solution, initial temperature, perturbation mechanisms, solution acceptance criterion, cooling schedule, termination criterion and additional settings such as the temperature reheating [74]. The pseudocode for SA is presented in Algorithm 3.

**Algorithm 3. *Pseudocode of SA***

**Input:** a problem instance, a solution search space (Neighborhood N), an initial solution $e_{\text{0}}$, control parameters

**Output:** The best solution $e^{*}$ found

1. best solution $e^{*}\leftarrow$ current solution $e^{\prime }\leftarrow$ initial solution $e_{\text{0}}$.

2. set temperature to the initial temperature $T^{sa}\leftarrow T_{\text{0}}^{sa}$.

3. set iteration k=0

4. **While** iteration k≤I **do**

5.  Choose a solution $e_{i+\text{1}}$ from the Neighborhood N according to perturbation mechanisms.

6.  **If** $e_{k+\text{1}}$ meets acceptance criterion **then**

7.   $e^{*}\leftarrow e_{k+\text{1}}$

**8.**  **End**

**9.**  **If** termination criterion is not met **then**

10.   update temperature according to cooling schedule; update parameters according to specific settings; k←k+1

**11.**  **End**

**12.** **End**

**13.** **Return** $e^{*}$

The initial solution is obtained using the new method described in Section 2.3. The selection of the other components is discussed in detail in the following sections.

### 3.1 Parameter selections

The initial temperature is computed based on the characteristics of the objective function, as specified in Equation (29), proposed by Aarts and Korst [75].


$$T_{\text{0}}^{sa}=\frac{\Delta_{E_{max}}}{\text{ln}(u)}$$ (29)

where $\Delta_{E_{max}}$ is the maximum expected change in objective value, and u is a constant. We generate 20 solutions $\left\{e_{k}\right\},k=\text{1},\cdots ,\text{2}\text{0}$ at the beginning of the search process and compute the corresponding objective function value $obj\left(e_{i}\right)$ for each solution. Then, $\Delta_{E_{max}}$ is the difference between the maximum and minimum objective values, i.e., $\Delta_{E_{max}}=max\left(obj\left(e_{k}\right)\right)-min\left(obj\left(e_{k}\right)\right).$ Additionally, the value of u is within the range of 1–5 empirically [76] but it can be adjusted based on acceptance probability $p_{acc}$ under the assumption that the desired probability of accepting unfavourable solution is 80%. Hence, replacing Δ in Equation (28) with $-\Delta_{E_{max}}$ in Equation (29) forms Equation (30):


$$T_{\text{0}}^{sa}=\frac{\Delta_{E_{max}}}{\text{ln}(u)}=\frac{-\Delta }{\text{ln}\left(p_{acc}\right)}$$ (30)

Solving for $u=\frac{\text{1}}{p_{acc}}=\frac{\text{1}}{\text{8}\text{0}\%}=\text{1}.\text{2}\text{5}$.

Once the initial temperature $T_{\text{0}}^{sa}$ is determined, a cooling schedule is implemented to govern the temperature updates at each iteration, as presented in Equation (31).


$$T_{k+\text{1}}^{sa}=\frac{T_{k}^{sa}}{\text{1}+\theta T_{k}^{sa}}$$ (31)

where θ is the cooling factor, and the choice of θ should satisfy the relationship $\text{1}+\theta T_{k}^{sa}>T_{k}^{sa}$ to guarantee eventual convergence. After conducting experiments on various θ it was decided that θ=0.000015 is used.

### 3.2 Perturbation mechanisms

One of the main challenges of SA is its tendency to get stuck in local minima. Therefore, an effective perturbation mechanism is essential for continuously improving the best-found solution [77]. In this study, perturbations are categorised into three types: mining-based, destination-based and grade-based perturbations, with the goal of advancing the mining and processing of ore blocks earlier while meeting all required constraints. At each iteration, a random number from {0, 1, 2} determines the perturbation type: 0 for mining-based, 1 for destination-based, and 2 for grade-based. Once selected, up to 10 feasible swaps are performed under the chosen type. Each type is described in detail below.

#### 3.2.1 Mining-based Perturbation.

This perturbation involves swapping mined materials in two blocks to ensure that those with higher grades are mined earlier while lower-grade or waste blocks are scheduled for later periods. Given the current solution $e_{k}$, precompute the movable periods for a single block or a group of blocks based on the mining status of their preceding and succeeding blocks. Specifically, the range of mining periods are determined by the latest mining period of the precedent blocks and the earliest mining period of the succeeding blocks:


$${RangeT}_{b}=\left[\underset{t}{argmax}\left(\;y_{b^{\prime }mt}|b^{\prime }{\in \Pi }_{b}\right),\underset{t}{argmin}\left(\;y_{b^{\prime \prime }mt}|b{\in \Pi }_{b^{\prime \prime }}\right)\right]$$ (32)

**Fig 2:**
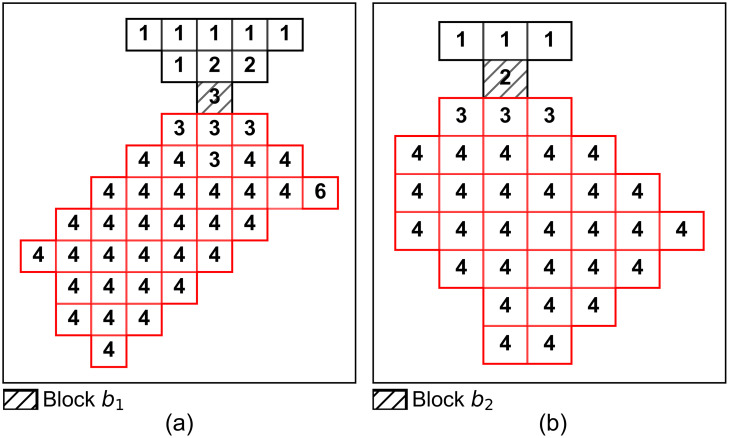
Section view of the mining-based perturbation for swapping the mining periods of blocks $b_{1}$ and $b_{2}$.

Fig 2 presents the potential swapping between blocks $b_{\text{1}}$ and $b_{\text{2}}$, based on the range of mining periods calculated using Equation (32), as depicted in section views. The parameter ${sw}_{b_{\text{1}},b_{\text{2}}}$ indicates whether a swap between blocks $b_{\text{1}}$ and $b_{\text{2}}$ occurs, either by their undiscounted values or by a probability p similar to the acceptance probability $p_{acc}$ in Equation (28).

To evaluate the swapping condition, generate a random decimal number from a uniform distribution, i.e., $u_{b_{\text{1}},b_{\text{2}}}\sim U(\text{0},\text{1})$. For cases where the mining period of blocks $b_{\text{2}}$ is later than that of block $b_{\text{1}}$ (i.e., $T_{b_{\text{1}}}<\;T_{b_{\text{2}}}$), the swapping status ${sw}_{b_{\text{1}},b_{\text{2}}}$ is defined as:


$${sw}_{b_{\text{1}},b_{\text{2}}}=\left\{ \begin{array}{cc} \text{1} & \sum_{j}v_{b_{\text{1}},j}x_{b_{\text{1}},j}<\sum_{j}v_{b_{\text{2}},j}x_{b_{\text{2}},j}\;or\;u_{b_{\text{1}},b_{\text{2}}}<p\\ \text{0} & otherwise \end{array}\right.\;$$ (33)

According to Equation (33), two blocks are swapped if the later-mined block has a higher value, or, with a small probability (p=5%), even if it is lower. accommodates hard constraints, such as blending requirements or the full encapsulation of PAF, which may require delaying or prioritizing certain blocks during mining.

**Fig 3:**
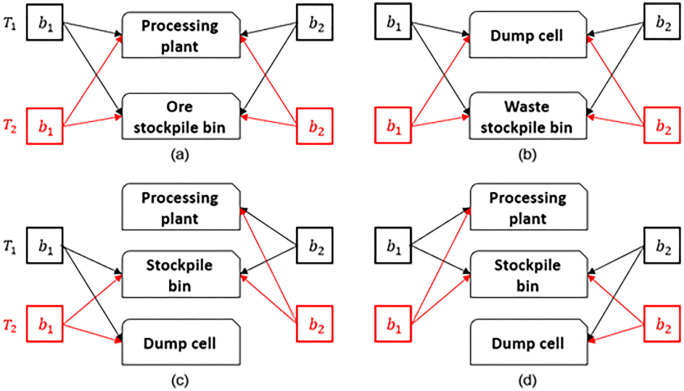
Graphical representations of different perturbations.


In addition, to ensure compliance with other constraints, the mined quantities of blocks $b_{\text{1}}$ and $b_{\text{2}}$ are redistributed based on their processing destinations. A block sent to a processing plant is classified as an ore block, while a block sent to a waste dump is regarded as a waste block. Fig 3 depicts several swaps between different material flowing destinations, where block $b_{\text{1}}$ is mined in period $T_{\text{1}}$, earlier than block $b_{\text{2}}$, that is mined in period $T_{\text{2}}$. It illustrates various swapping scenarios, including the exchange of materials between two ore blocks in Fig 3a, two waste blocks in Fig 3b, later-mined ore materials with earlier-mined waste materials in Fig 3c, which may involve rescheduling waste materials from the waste stockpile to compensate for the moved waste materials in the earlier period, and later-mined waste materials with earlier-mined ore materials in Fig 3d. Note that Fig 3d is not considered in the mining-based perturbation but rather in the grade-based perturbation discussed in Section 3.2.3, as postponing a high-grade ore block in favor of a low-grade waste block to a later period is allowed within a grade range to reduce the impact of profit loss. To summarise, the changes in mined quantities of materials in blocks associated with Fig 3a – Fig 3c, are formulated in Equations (34) – (37). More specifically, Equation (34) represents the exchange of materials between two ore blocks ($b_{\text{1}}$ and $b_{\text{2}}$), Equation (35) represents the exchange of materials between two waste blocks ($b_{\text{1}}$ and $b_{\text{2}}$), and Equation (36) represents the exchange of materials between an earlier mined waste blocks $b_{\text{1}}$ and a later mined ore block $b_{\text{2}}$. The parameter $R_{j}^{t}$ represents the remaining capacity at processing destination j during mining period t in the current solution e.

$$\left\{\begin{array}{c} b_{\text{1}}\left\{ \begin{array}{c} T_{\text{1}}:q_{\text{1}j}^{T_{\text{1}}}:=\text{0}\;j\in \left\{P,S\right\}\\ T_{\text{2}}:\text{0}:=\left\{ \begin{array}{cc} \left(\sum_{j^{\prime }\in \left\{P,S\right\}}q_{\text{1}j^{\prime }}^{T_{\text{1}}}-R_{j}^{T_{\text{2}}}\right)\cdot \text{1}\left(R_{j}^{T_{\text{2}}}\geq \sum_{j^{\prime }\in \left\{P,S\right\}}q_{\text{1}j^{\prime }}^{T_{\text{1}}}\right)+R_{j}^{T_{\text{2}}} & j\in \left\{P\right\}\\ \left(\sum_{j^{\prime }\in \left\{P,S\right\}}q_{\text{1}j^{\prime }}^{T_{\text{1}}}-R_{j}^{T_{\text{2}}}\right)\cdot \text{1}\left(R_{j}^{T_{\text{2}}}<\sum_{j^{\prime }\in \left\{P,S\right\}}q_{\text{1}j^{\prime }}^{T_{\text{1}}}\right)\; & j\in \left\{S\right\} \end{array}\right.\; \end{array}\right.\;\\ b_{\text{2}}\left\{ \begin{array}{c} T_{\text{1}}:\text{0}:=\left\{ \begin{array}{cc} \left(\sum_{j^{\prime }\in \left\{P,S\right\}}q_{\text{2}j^{\prime }}^{T_{\text{2}}}-R_{j}^{T_{\text{1}}}\right)\cdot \text{1}\left(R_{j}^{T_{\text{1}}}\geq \sum_{j^{\prime }\in \left\{P,S\right\}}q_{\text{2}j^{\prime }}^{T_{\text{2}}}\right)+R_{j}^{T_{\text{1}}} & j\in \left\{P\right\}\\ \left(\sum_{j^{\prime }\in \left\{P,S\right\}}q_{\text{2}j^{\prime }}^{T_{\text{2}}}-R_{j}^{T_{\text{1}}}\right)\cdot \text{1}\left(R_{j}^{T_{\text{1}}}<\sum_{j^{\prime }\in \left\{P,S\right\}}q_{\text{2}j^{\prime }}^{T_{\text{2}}}\right)\; & j\in \left\{S\right\} \end{array}\right.\;\\ T_{\text{2}}:q_{\text{2}j}^{T_{\text{2}}}:=\text{0}\;j\in \left\{P,S\right\} \end{array}\right.\; \end{array}\right\}$$ (34)

$$\left\{\begin{array}{c} b_{\text{1}}\left\{ \begin{array}{c} T_{\text{1}}:q_{\text{1}j}^{T_{\text{1}}}:=\left\{ \begin{array}{cc} \left(q_{\text{1}j}^{T_{\text{1}}}-q_{\text{2}j}^{T_{\text{2}}}\right)\cdot \text{1}\left(q_{\text{2}j}^{T_{\text{2}}}\leq q_{\text{1}j}^{T_{\text{1}}}\right)\; & j\in \left\{D\right\}\\ \text{0}\; & j\in \left\{S\right\} \end{array}\right.\;\\ T_{\text{2}}:\text{0}:=\left\{ \begin{array}{cc} q_{\text{2}j}^{T_{\text{2}}}\cdot \text{1}\left(q_{\text{2}j}^{T_{\text{2}}}\leq q_{\text{1}j}^{T_{\text{1}}}\right)+q_{\text{1}j}^{T_{\text{1}}}\cdot \text{1}\left(q_{\text{2}j}^{T_{\text{2}}}>q_{\text{1}j}^{T_{\text{1}}}\right)\; & j\in \left\{D\right\}\\ q_{\text{1}j}^{T_{\text{1}}} & j\in \left\{S\right\} \end{array}\right.\; \end{array}\right.\;\\ b_{\text{2}}\left\{ \begin{array}{c} T_{\text{1}}:\text{0}:=\left\{ \begin{array}{cc} q_{\text{2}j}^{T_{\text{2}}}\cdot \text{1}\left(q_{\text{2}j}^{T_{\text{2}}}\leq q_{\text{1}j}^{T_{\text{1}}}\right)+q_{\text{1}j}^{T_{\text{1}}}\cdot \text{1}\left(q_{\text{2}j}^{T_{\text{2}}}>q_{\text{1}j}^{T_{\text{1}}}\right)\; & \;j\in \left\{D\right\}\\ \left(q_{\text{2}j}^{T_{\text{2}}}-q_{\text{1}j}^{T_{\text{1}}}\right)\cdot \text{1}\left(q_{\text{2}j}^{T_{\text{2}}}>q_{\text{1}j}^{T_{\text{1}}}\right) & \;j\in \left\{S\right\} \end{array}\right.\;\\ T_{\text{2}}:q_{\text{2}j}^{T_{\text{2}}}:=\left\{ \begin{array}{cc} \left(q_{\text{2}j}^{T_{\text{2}}}-q_{\text{1}j}^{T_{\text{1}}}\right)\cdot \text{1}\left(q_{\text{2}j}^{T_{\text{2}}}>q_{\text{1}j}^{T_{\text{1}}}\right)\; & j\in \left\{D\right\}\\ \text{0}\; & j\in \left\{S\right\} \end{array}\right.\; \end{array}\right.\; \end{array}\right\}$$ (35)

$$\left\{\begin{array}{c} b_{\text{1}}\left\{ \begin{array}{c} T_{\text{1}}:q_{\text{1}j}^{T_{\text{1}}}:=\text{0}\;j\in \left\{D,S\right\}\\ T_{\text{2}}:\text{0}:=\left\{ \begin{array}{cc} \text{0}\; & j\in \left\{D\right\}\\ \sum_{j^{\prime }\in \left\{D,S\right\}}q_{\text{1}j^{\prime }}^{T_{\text{1}}}\; & j\in \left\{S\right\} \end{array}\;\right.\; \end{array}\right.\\ b_{\text{2}}\left\{ \begin{array}{c} T_{\text{1}}:\text{0}:=\left\{ \begin{array}{cc} \text{1}\left(R_{j}^{T_{\text{1}}}\geq \sum_{j^{\prime }\in \left\{P,S\right\}}q_{\text{2}j^{\prime }}^{T_{\text{2}}}\right)\left(\sum_{j^{\prime }\in \left\{P,S\right\}}q_{\text{2}j^{\prime }}^{T_{\text{2}}}-R_{j}^{T_{\text{1}}}\right)+R_{j}^{T_{\text{1}}} & j\in \left\{P\right\}\\ \text{1}\left(R_{j}^{T_{\text{1}}}<\sum_{j^{\prime }\in \left\{P,S\right\}}q_{\text{2}j^{\prime }}^{T_{\text{2}}}\right)\left(\sum_{j^{\prime }\in \left\{P,S\right\}}q_{\text{2}j^{\prime }}^{T_{\text{2}}}-R_{j}^{T_{\text{1}}}\right)\; & j\in \left\{S\right\} \end{array}\;\right.\;\\ T_{\text{2}}:q_{\text{2}j}^{T_{\text{2}}}:=\text{0}\;j\in \left\{P,S\right\} \end{array}\right.\; \end{array}\right\}$$ (36)

#### 3.2.2 Destination-based Perturbation.

This type of perturbation aims to increase the likelihood of processing high-grade materials in ore stockpiles earlier while reducing the need for rehandling NAF waste materials from waste stockpiles to waste dumps. The grade of materials stored in a stockpile bin is represented by the bin’s average grade. Hence, considering periods t>1, ore materials are retrieved from stockpiles to processing plants in descending order of average grade when the processing capacity is not full. Otherwise, blocks designated for processing are exchanged with materials from a stockpile bin if their grade

falls within the bin’s range but is lower than the average grade, i.e., $g_{b}\in (g_{s,low}\;,g_{s,high}]$, and $g_{b}<g_{s}$. On the other hand, the rehandling of NAF waste from waste stockpiles typically occurs in the final period when the amount of NAF waste mined directly from pits is insufficient to cover the waste dumping requirements. In this regard, material from blocks sent to waste stockpiles is randomly selected and rescheduled to waste dump cells originally filled by rehandled waste, while satisfying the dumping precedence constraints.

#### 3.2.3 Grade-based Perturbation.

Similar to Kumral [59], the grade-based perturbation modifies block classification. That is, ore-type blocks processed at processing plants are instead transported to waste dumps as a waste-type block, and vice versa, as shown in Fig 3d. To control the impact, the grade of ore blocks selected for exchange is restricted by a predefined upper threshold. This reclassification can alter the effective cut-off grade, thereby directly improving NPV. For example, converting a marginal ore block to waste may enable earlier access to higher-grade ore, enhancing overall value. Conversely, reclassifying waste as ore can improve resource utilization. The corresponding quantity exchanges for this perturbation are defined in Equation (37) visualised in Fig 3d, i.e., the exchange of materials between an earlier mined ore blocks $b_{\text{1}}$ and a later mined waste block $b_{\text{2}}$.


$$\left\{\begin{array}{c} b_{\text{1}}\left\{ \begin{array}{c} T_{\text{1}}:q_{\text{1}j}^{T_{\text{1}}}:=\text{0}\;j\in \left\{P,S\right\}\\ T_{\text{2}}:\text{0}:=\left\{ \begin{array}{cc} \text{1}\left(R_{j}^{T_{\text{2}}}\geq \sum_{j^{\prime }\in \left\{P,S\right\}}q_{\text{1}j^{\prime }}^{T_{\text{1}}}\right)\left(\sum_{j^{\prime }\in \left\{P,S\right\}}q_{\text{1}j^{\prime }}^{T_{\text{1}}}-R_{j}^{T_{\text{2}}}\right)+R_{j}^{T_{\text{2}}} & j\in \left\{P\right\}\\ \text{1}\left(R_{j}^{T_{\text{2}}}<\sum_{j^{\prime }\in \left\{P,S\right\}}q_{\text{1}j^{\prime }}^{T_{\text{1}}}\right)\left(\sum_{j^{\prime }\in \left\{P,S\right\}}q_{\text{1}j^{\prime }}^{T_{\text{1}}}-R_{j}^{T_{\text{2}}}\right)\; & j\in \left\{S\right\} \end{array}\;\right.\; \end{array}\right.\;\\ b_{\text{2}}\left\{ \begin{array}{c} T_{\text{1}}:\text{0}:=\left\{ \begin{array}{cc} \;\text{0}\; & \;j\in \left\{D\right\}\\ \sum_{j^{\prime }\in \left\{P,S\right\}}q_{\text{2}j^{\prime }}^{T_{\text{1}}} & \;j\in \left\{S\right\} \end{array}\;\right.\;\\ T_{\text{2}}:q_{\text{2}j}^{T_{\text{2}}}:=\text{0}\;j\in \left\{D,S\right\} \end{array}\right.\; \end{array}\right\}$$ (37)

### 3.3 Temperature Reheating

Temperature reheating refers to resetting the temperature value to a higher one to trigger effective escapes from local optima [68]. Here, reheating is performed when no new solution has been accepted in the last L candidate moves, using the standard formula $T_{i+\text{1}}^{sa}=\frac{T_{i}^{sa}}{\delta },\;\text{0}<\delta <\text{1}$, as discussed and implemented by Abramson [78]. This mechanism raises the temperature, enabling the algorithm to adopt a more exploratory search behaviour. Meanwhile, the searching process gradually converges when no better solution can be found after multiple times of reheating operation. Hence, five times of consecutive reheating was used as the stopping criteria. In addition, to ensure sufficient exploitation within the neighbourhood L=45 and δ=0.7 were decided through extensive experimentation with various combinations.

## 4 Case studies

This section presents the application of the mathematical model and the proposed solution procedures—including the variable reduction method, a high-value search heuristic for generating the initial feasible solution, and the simulated annealing (SA)-based algorithm—at an operational open-pit mining complex in the Eastern Goldfields of Western Australia. The mining complex comprises two mines and two processing streams: a Carbon-in-Leach (CIL) process for relatively high-grade ores and a heap leach process for relatively low-grade ores. It also includes ore stockpiles divided into 42 grade bins, three waste stockpiles for NAF waste rocks, and a waste dump that accommodates both NAF and PAF wastes in dedicated dump cells. The new methods have been implemented in two separate cases, Case A and Case B.

Tables 1–3 present the block model, waste dump configuration, economic technical, and operational parameters applicable to both Cases A and B.

**Table 1:** Block model and waste dump parameters.

	Case A		Case B	
	Pit 1	Pit 2	Pit 3	Pit 4
Block dimensions (m)	10x20x5	20x20x5	10x20x5	20x20x5
Number of blocks	1,800	341	3,604	4,536
Number of PAF blocks	138	16	161	247
Maximum grade	2.471	1.392	3.105	3.093
Number of dump cells	68		155	
Number of dump layers	3		3	
Dump cell dimensions (m)	20x20x10		40x40x20	
Number of PAF cells	6		34	
Number of periods (years)	3		6	

**Table 2:** Economic and technical parameters.

Economic Parameters	Value
Gold price ($ per gram of Au)	22.503
Refining cost ($ per gram of Au)	1
Mining cost ($ per tonne of material)	1 - 1.125
Heap leach processing cost ($ per tonne of ore)	3.75
Carbon-in-leach processing cost ($ per tonne of ore)	6.25
Heap leach processing recovery rate (%)	75
Carbon-in-leach processing recovery rate (%)	93
Haulage cost ($ per tonne-km)	0.075
Stockpile rehandling cost ($ per tonne of material)	0.125
Discount rate (%)	10

**Table 3:** Operational parameters (capacities per period).

Operational Capacities	Case A		Case B	
	Pit 1	Pit 2	Pit 3	Pit 4
Mining capacity (tonnes of material per period)	400,000	200,000	800,000	2,000,000
Heap leach capacity (tonnes of ore per period)	100,000		400,000	
Carbon-in-leach capacity (tonnes of ore per period)	50,000		800,000	

All experiments for implementation of the proposed procedures were conducted on an AWS cloud platform [79] where a Python 3.9 environment was set up on an Ubuntu virtual machine. The machine was configured with 122 GB of RAM, 32 vCPUs, and 100 GB of storage. However, to evaluate the performance of the proposed solution methods through valid comparisons, both cases were also solved using the commercial solver CPLEX [80] that generates optimal solutions within a defined gap.

Table 4 presents the efficiency of variable reduction procedure given in Section 2.2. By eliminating certain variables, such as those representing whether blocks are extracted, or dump cells are filled in specific periods. In addition, the variable reduction procedure allows removal of associated mining block and dump cell precedence constraints. This leads to a substantial reduction in both variables and constraints, particularly the continuous variables.

**Table 4:** Number of variables and constraints before and after applying the variable reduction technique.

	Case A (Number of Blocks = 2,141)			Case B (Number of Blocks = 8,140)		
	Number of Binary Variables	Number of Continuous Variables	Number of Constraints	Number of Binary Variables	Number of Continuous Variables	Number of Constraints
Before variable reduction	6,717	468,405	14,696	49,770	3,231,048	102,127
After variable reduction	5,079	134,346	13,332	35,177	964,981	95,468
Percent reduction	24.4	71.3	9.3	29.3	70.1	6.5

Table 5 presents the application of the HVS heuristic for generating the initial feasible solution, which is subsequently used in the SA algorithm. The table also includes results from the CPLEX solver to illustrate the performance of the proposed procedures. As shown in Table 5, for Case A, the SA-based algorithm requires 97.3 minutes, which is significantly shorter than the 558.5 minutes taken by CPLEX. In terms of solution quality, the net present value (NPV) achieved by the SA-based algorithm is only 6.08% lower than that obtained by CPLEX, whose solution has an optimality gap within 1%.

**Table 5:** Performance evaluation of the proposed algorithm compared to the exact method.

Solution Method	Case A (Number of Blocks = 2,141)			Case B (Number of Blocks = 8,140)		
	NPV (Million $)	Running time (mins)	Optimality gap (%)	NPV (Million $)	Running time (mins)	Optimality gap (%)
SA-based	13.44	97.3	6.08%	104.02	292.3	–
CPLEX	14.31	558.5	0.88%	–	–	–

Table 6 and Table 7 present the production plans generated by CPLEX and the proposed SA-based algorithm, respectively. The NPV difference primarily stems from a higher cut-off grade in the first two years under the SA approach, resulting in less ore sent to the processing plants and ore stockpiles. Additionally, the SA-based algorithm incurs a waste rehandling cost for relocating 2,850 tonnes of NAF waste. While the SA algorithm processes 28,200 tonnes less ore as compared to the CPLEX, it handles 6,110 tonnes less waste. As a result, the overall stripping ratio (ratio of quantity of waste to the quantity of ore) is slightly higher for the SA-based algorithm (1.461) compared to CPLEX (1.354).

**Table 6:** Production plan produced by the exact method for Case A.

Periods	From Mines (tonnes)				From Ore Stockpiles (tonnes)	From Waste Stockpiles (tonnes)	Cut-off grade
**To Processing Streams**	**To Ore Stockpiles**	**To Waste Stockpiles**		**To Waste dump**	**To Processing Streams**	**To Waste dump**
1	150,000	19,850	15,905	98,125	–	–	0.290
2	72,700	–	31,500	72,537	19,850	–	0.288
3	85,775	–	34,790	181,713	–	–	0.286
Total	308,475	19,850	82,195	362,375	19,850	–	

**Table 7:** Production plan produced by the SA-based algorithm for Case A.

Periods	From Mines (tonnes)				From Ore Stockpiles (tonnes)	From Waste Stockpiles (tonnes)	Cut-off grade
	To Processing Streams	To Ore Stockpiles	To Waste Stockpiles		To Waste dump	To Processing Streams	To Waste dump
1	150,000	8,500	21,035	100,150	–	–	0.297
2	58,800	–	53,575	81,100	8,500	–	0.295
3	82,825	–	–	182,600	–	2,850	0.283
Total	291,625	8,500	74,610	363,850	8,500	2,850	

**Fig 4:**
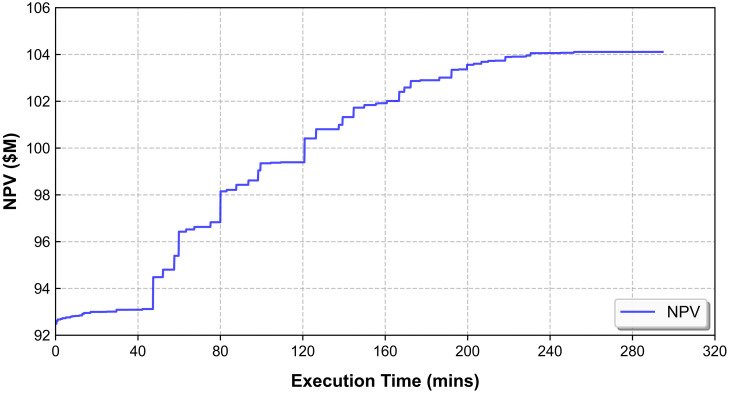
Evolution of NPV over execution time.

As shown in Table 5, CPLEX fails to generate an optimal solution for Case B within a computation time exceeding 9 hours, ultimately resulting in an out-of-memory error. In contrast, the SA-based algorithm delivers the best or an improved solution within 292.3 minutes. Fig 4 illustrates the evolution of the net present value (NPV), with the SA-based algorithm achieving a significant 12.6% increase compared to the initial value. This also highlights the effectiveness of the High-Value Search (HVS) heuristic, which enabled the SA-based algorithm to converge to the best solution within 292.3 minutes. Given this strong performance in Case B, which involves a larger and more complex dataset, the subsequent analysis will focus on the results obtained using the SA-based algorithm for Case B.

**Fig 5:**
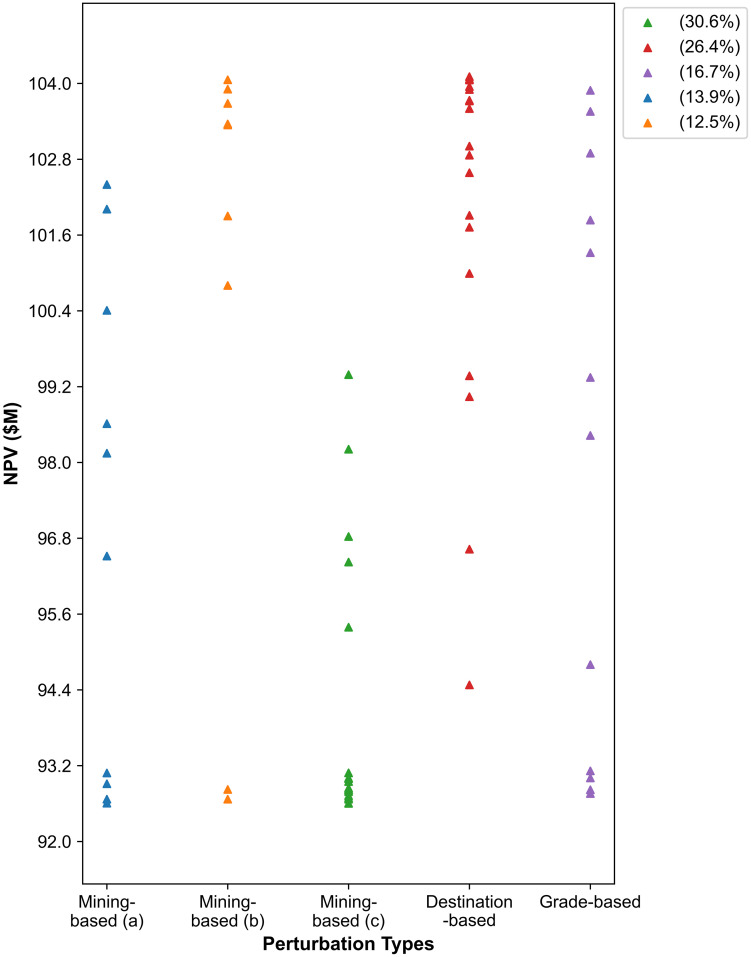
The distribution of NPV improvement instances across different perturbation types.

The NPV improvements associated with each type of perturbation are presented in Fig 5. The three sub-types of the mining-based perturbation—(a), (b), and (c), as described in Section 3.2—are illustrated individually. Among the five perturbation types, sub-type (c), which prioritises the early extraction of high-grade ores while delaying waste removal, accounts for 31% of the improvement instances. This highlights its leading performance in achieving the most frequent NPV increases.

This perturbation proves particularly effective during the early to mid-stages of the search process, when there is a higher probability of replacing low-grade blocks with high-grade ones. As the search progresses and the number of beneficial swaps decreases, the destination-based perturbation becomes more influential during the mid-to-late stages. It enhances NPV by increasing the likelihood that processing plants receive higher-grade ores from stockpiles rather than directly from the mines, and by maximising the direct allocation of NAF waste to the waste dump—thereby reducing rehandling operations. In contrast, the remaining perturbation types have a less significant impact on NPV improvement, as reflected by their lower proportions.

**Fig 6:**
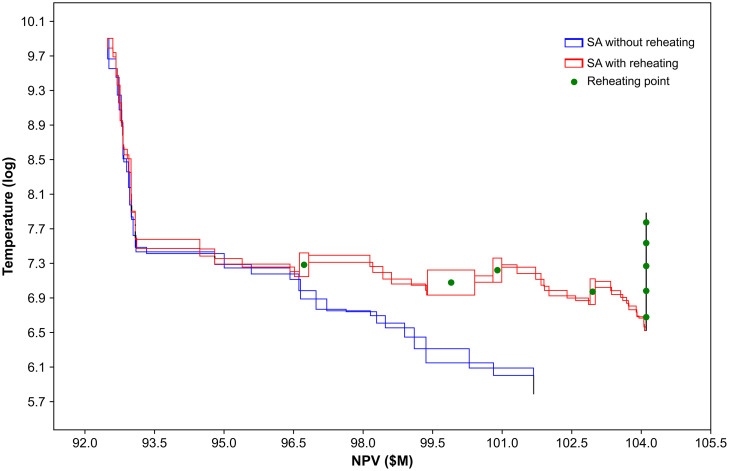
Change in temperature with improvement in NPV.

The impact of temperature reheating on NPV is illustrated in Fig 6, demonstrating how temperature evolves with each NPV increase, both with and without the reheating applied under the exact same perturbation types at each iteration. In the case of the proposed SA-based algorithm without reheating, the process stops after 300 iterations with no improvement in the solution. It can be observed that the NPV improvements remain nearly identical before the application of reheating, and there is also no noticeable difference during the first two reheating stages. However, the temperature has been maintained and elevated above 800 during the first four reheating operations, which helps to revive the search process and enhance exploration. This effect is evident in the increased frequency of NPV improvements after the third reheating, in contrast to the scenario without reheating, where the temperature gradually falls below 700, limiting further exploration. Nevertheless, reheating becomes ineffective when the temperature drops below 650 after the fourth time of reheating, suggesting that the algorithm may have converged to a locally sub-optimal solution. Overall, the proposed SA-based algorithm with reheating achieves a $2.43 million higher NPV compared to the version without reheating.

Table 8 and Table 9 compare the material flow before and after applying the proposed SA-based algorithm. From Table 8, the initial solution obtained using HVS heuristic in Section 2.3 directs a total of 6,557,355 tonnes of ore to processing streams, along with 13,200 and 123,000 tonnes of low-to-medium grade of ore to ore stockpiles during periods 1 and 5, respectively, which are later processed in periods 2 and 6. For waste materials, 695,150 tonnes are transported directly from mines to waste stockpiles, while 7,063,200 tonnes are sent to the waste dump. However, 12,000 tonnes of waste are rehandled from waste stockpiles to the waste dump in period 6 due to the PAF full encapsulation requirement, incurring an additional $44,726.4 in rehandling costs.

**Table 8:** Material flows produced by the initial solution (HVS heuristic).

Periods	From Mines (tonnes)				From Ore Stockpiles (tonnes)	From Waste Stockpiles (tonnes)
	To Processing Streams	To Ore Stockpiles	To Waste Stockpiles	To Waste dump	To Processing Streams	To Waste dump
1	1,200,000	13,200	63,615	646,385	–	–
2	1,020,000	–	154,635	1,571,215	13,200	–
3	1,132,000	–	147,170	1,495,330	–	–
4	1,096,000	–	112,270	1,140,730	–	–
5	1,200,000	123,000	107,250	1,089,750	–	–
6	909,355	–		1,119,790	123,000	12,000
Total	6,557,355	136,200	584,940	7,063,200	136,200	12,000

**Table 9:** Material flows produced by the proposed SA-based algorithm.

Periods	From Mines (tonnes)				From Ore Stockpiles (tonnes)	From Waste Stockpiles (tonnes)
	To Processing Streams	To Ore Stockpiles	To Waste Stockpiles	To Waste dump	To Processing Streams	To Waste dump
1	1,200,000	86,400	7,830	628,170	–	–
2	1,085,000	–	20,480	1,643,020	86,400	–
3	1,191,000	–	19,575	1,570,425	–	–
4	1,200,000	33,500	13,705	1,099,295	–	–
5	1,200,000	147,100	13,310	1,067,690	–	–
6	1,015,250	–	–	1,034,600	180,600	5,200
Total	6,891,250	267,000	74,900	7,043,200	267,000	5,200

In contrast, after running the proposed SA-based algorithm, the material flow in Table 9 shows that relatively higher quantity of ore and lower quantity of waste is produced. The quantity of ore extracted from mines and retrieved from the ore stockpiles increases by 333,895 and 130,800 tonnes, respectively, compared to the initial solution. On the other hand, while the waste sent directly to the waste dump decreases only slightly by 20,000 tonnes, the waste transported to stockpiles is significantly reduced by 510,040 tonnes, resulting in a $2,413,983.95 cost reduction in waste dumping. Moreover, the waste rehandling cost drops to $20,055.62, with only 5,200 tonnes of waste being rehandled.

**Fig 7:**
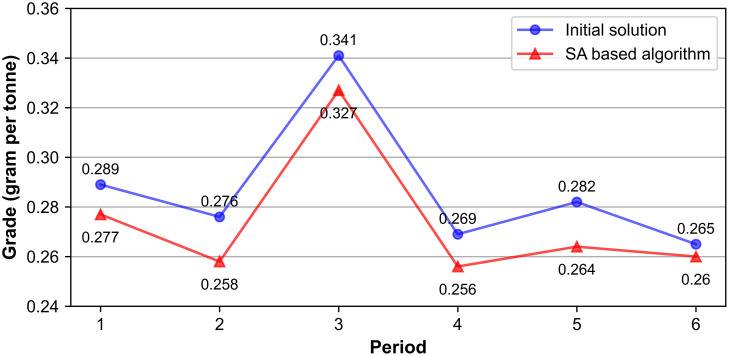
Cut-off grade in each period before and after SA.

These improvements stem from the proposed SA-based algorithm’s ability to detect a lower cut-off grade in each period, enabling the processing of more ore while reducing waste, which is also observed evidently in Fig 7. Specifically, the cut-off grade in the SA schedule decreases by 0.005–0.018 grams per tonne across periods compared with the initial solution. As a result, blocks with grades between the two cut-off thresholds are treated as ore rather than waste, consistent with the trends shown in Table 8 and Table 9, where more material is directed to processing plants and less to waste dumps. Although the average feed grade under SA is marginally lower (0.274 vs. 0.288 grams per tonne), the total gold recovered over the life of mine increases, yielding a higher NPV. Importantly, this strategy also satisfies the PAF encapsulation requirement while simultaneously reducing rehandling costs.

## 5. Conclusions

This paper presents an innovative Simulated Annealing (SA)-based metaheuristic procedure for simultaneous optimisation of block extraction and waste dump scheduling in open pit mining complexes. The proposed method generates efficient and effective solutions for relatively large instances of a mining system that integrates multiple mines, processes, stockpiles, and waste dumps allowing a responsible disposal of PAF wastes through encapsulation within NAF wastes. Therefore, unlike some of the best studies available in the literature, the proposed method not only offers solution to more accurate representation of real-world scenarios in open pit mining complexes, but also promotes sustainable mining practices through responsible disposal of wastes, which is often difficult to achieve using existing mathematical formulations due to the additional complexity and volume of constraints that comes with waste dump scheduling.

The proposed approach incorporates several advanced and innovative mechanisms, including a variable deduction technique, an efficient heuristic for initial solution, and then custom-designed perturbations along with a temperature reheating operation within SA algorithm that ensures significant improvements in the initial solution.

The choice of simulated annealing (SA) offers the flexibility required to explore high-quality solutions within a reasonable computation time. Through numerical experiments on two datasets (Cases A and B), the proposed SA-based algorithm demonstrates strong performance—matching the solution quality of CPLEX for the smaller dataset while achieving the solution in significantly less time (82% reduction), with only a 6.08% difference in NPV. With Case A confirming the efficiency of the proposed procedures, the results for the larger dataset highlight the substantial contributions of both perturbation strategies and reheating mechanisms in enhancing the overall NPV.

Notably, two specific perturbation strategies play a key role in this improvement: (1) shifting low-to-medium grade blocks from earlier periods to later ones in favour of high-grade blocks during the early stages of the search process, and (2) prioritising the allocation of waste to the dump rather than to stockpiles in the later periods. These targeted adjustments enable the algorithm to achieve more effective scheduling outcomes.

Future research could explore the development and implementation of alternative metaheuristic methods to compare with the proposed SA-based approach. Additionally, while this study focuses on a deterministic framework to demonstrate the effectiveness of the proposed SA-based approach with integrated waste-dumping and PAF encapsulation requirements, we acknowledge that real-world mine planning is subject to both geological and economic uncertainty as well as evolving operational conditions. Future extensions will therefore consider (i) incorporating stochastic programming and robust optimisation to capture variability in ore grades, costs, and market prices, and (ii) implementing a dynamic rescheduling mechanism that periodically updates production plans as new data becomes available. Together, these developments would enable more adaptive and resilient mine planning and move the framework closer to digital twin applications in practice.
